# Expression of HIF-1α and HIF-2α correlates to biological and clinical significance in papillary thyroid carcinoma

**DOI:** 10.1186/s12957-016-0785-9

**Published:** 2016-02-04

**Authors:** Yan-Mei Liu, Shen-Peng Ying, Ying-Rui Huang, Yin Pan, Wei-Jun Chen, Ling-Qin Ni, Jin-Ye Xu, Qin-Yan Shen, Yong Liang

**Affiliations:** 1 Department of Radiation Oncology, Taizhou Central Hospital, Taizhou, 318000 Zhejiang China; 2 Taizhou University, Taizhou, 318000 Zhejiang China; 3 Department of Breast and Thyroid Surgery, Taizhou Central Hospital, Taizhou, 318000 Zhejiang China; 4 Department of Breast and Thyroid Surgery, Taizhou Municipal Hospital, Taizhou, 318000 Zhejiang China; 5 Department of Wenzhou Medical University, Wenzhou, 325000 Zhejiang China; 6 Department of The First Affiliated Hospital of Wenzhou Medical University, Wenzhou, 325000 Zhejiang China

**Keywords:** HIF-1α, HIF-2α, RT-PCR, IHC, Western blot

## Abstract

**Background:**

The aim of this study was to detect the expression of hypoxia-inducible factor (HIF)-1α and HIF-2α in papillary thyroid carcinoma (PTC) compared with normal thyroid tissues.

**Methods:**

The mRNA levels and protein levels of HIF-1α and HIF-2α were detected by real-time PCR and Western blot separately in 30 pairs of PTCs and normal thyroid cases. The protein levels were also detected by immunohistochemistry (IHC) using 92 samples of PTC group and 46 normal samples as control group for analyzing the biological and clinical significance of the expression of HIF-1α/HIF-2α.

**Results:**

Real-time PCR results showed the mRNA level of *HIF*-1α and HIF-2α were significantly higher in PTC than normal group (*P* < 0.001). Also, significantly higher positive rates (73 %/65 %) of HIF-1α and HIF-2α were observed in PTC compared with the control group (27 %/35 %) by IHC (*P* < 0.01); the consistent results were gotten with Western blot. Although we did not find a significant correlation between the expression of HIF-1α and HIF-2α with gender, age, calcification, or Hashimoto’s disease in the present study (*P* > 0.05), both of their expressions were correlated to lymph node metastasis (*P* < 0.05), capsular invasion (*P* < 0.05), and TNM stage (*P* < 0.05).

**Conclusions:**

Overexpression of HIF-1α and HIF-2α are associated with the carcinogenesis of PTC, served as potential biomarkers of PTC.

## Background

As one of the most common endocrine tumor, thyroid cancer has become the fifth highest incidence of malignant tumor among American women [1]. The incidence of thyroid cancer, especially papillary thyroid carcinoma (PTC), was increased constantly over the recent years at a remarkable rate, averaging almost 4 % per year [2]. About 60,220 new cases were reported in 2013 [3] which have attracted much attention.

The PTC shows some special biological behaviors, such as it is easily accompanied with calcification or cystic in the development of tumor although the volume is small, and it is easily accompanied with lymph node metastasis (LNM) [4]. As we know, tumor growth requires the presence of a local vascular network that supplies both oxygen and nutrients to tumor cells. And most tumor cells have adapted to and can grow in a hypoxic condition. Such hypoxic zones have been postulated to increase patient treatment resistance and favor tumor progression [5, 6].

Hypoxia-inducible factor (HIF), known as a heterodimer transcription factor consisting of an oxygen-sensitive alpha subunit (HIF-α) and a constitutive beta subunit (HIF-β) [7], is closely associated with the formation of hypoxic microenvironment [8]. Under hypoxia, HIF-α subunits are stabilized and translocated to the nucleus where they heterodimerize with arylhydrocarbon receptor nuclear translocator (ARNT) and bind to hypoxia response elements (HREs) located within regulatory elements of HIF target genes. There are three types of HIF-α subunits, HIF-1α, HIF-2α, and HIF-3α, and at present, the HIF-1α and HIF-2α gained more attention [9–12]. Although it has been reported that HIF is associated with tumorigenesis [13] or radiosensitivity [14], reports on how HIF-1α and HIF-2α affect the oncogenesis showed inconsistent results [15, 16]. And overexpression of HIF-1α and HIF-2α correlated with different kinds of cancers, respectively [17, 18].

Thus, the aim of this study was to investigate the expression of HIF-1α and HIF-2α in papillary thyroid carcinoma and its roles in the development of PTC.

## Methods

### Patients and specimens

All the materials were obtained with agreement of patients and signed the informed consent. The usage of human materials for analysis was approved by the local ethical committee. All PTCs were staged according to the International Union Against Cancer (UICC) 2009 guidelines [19]. Ninety-two samples of PTC group (age range from 23 to 72 years, 58 male and 34 female) and control group (46 cases total, age range from 21 to 76 years, 19 male and 27 female) were collected from patients who underwent surgical resection at the Department of Tai Zhou Central Hospital between January to October in 2013, and the control samples were relatively normal tissues from the nodular goiters. All cases were confirmed by pathologists and divided into nine groups by age, sex, tumor size, etc. All tissue samples were obtained from the patients prior to any medical treatment.

### RNA extraction and first-strand cDNA synthesis

Of each sample, 50- to 100-mg tissues from 30 fresh frozen tumors and normal tissues, respectively, were used to isolate total RNA with TRIzol reagent (Invitrogen, Camarillo, CA, USA). All the processes of RNA extraction were done on ice, and all the workers wear masks and gloves to prevent the degradation of RNA. The amount and quality of the extracted RNA were determined by the Nano Drop Spectrophotometer (Thermo Scientific, Wilmington, DE, USA). The cDNA synthesis was performed under the condition of 37 °C for 60 min in a volume of 20 μL containing 2 μL RNA, 2 μL 10 × RT mix, 2 μL dNTP, 2 μL Oligo-dT15, 1 μL Quant Reverse Transcriptase, and 11 μL RNase-free ddH_2_O in reaction buffer.

### Real-time polymerase chain reaction

The primer sequences used herein were HIF-1α forward 5′-ACTTCTGGATGCTGGTGATTTG-3′, reverse 5′-GCTTCGCTGTGTGTTTTGTTCT-3′; HIF-2α forward 5′-TCATGCGACTGGCAATCAGC-3′, reverse 5′-GTCACCACGGCAATGAAACC-3′; and glyceraldehyde-3-phosphate dehydrogenase (GAPDH) forward 5′-CAAAGCTGGTGTGGGAGG-3′, reverse 5′-CTCCTGGAAGATGGTGATGG-3′. SYBR Green mix, cDNA template, 0.3 μL forward primer, 0.3 μL reverse primer, and ddH_2_O were added into the reaction system (20 μL total volume). All samples were amplified using the following standard PCR system: 95 °C pre-degeneration for 2 min, 95 °C degeneration for 15 s, 60 °C annealing for 30 s, and 68 °C extension for 60 s within 40 circulations. After reactions were completed, baseline and threshold were adjusted in the ABI 7500 software system (Applied Biosystem, USA) where the cycle threshold (CT) value of each reaction hole was read. This reaction has been repeated for three times. Data were analyzed according to the comparative CT value method and were normalized according to the GAPDH expression in each sample.

### Immunohistochemical analysis

One representative paraffin-embedded block from each case was selected and sectioned serially at 4 μm. Slides were baked at 68 °C for 2 h and soaked in xylene; antigen retrieval was performed by boiling the slides with citrate puffer (pH = 6) at 95 °C, then blocked by normal goat serum. The samples were incubated with an antibody against HIF-1α (1:100; rabbit monoclonal, clone; ab51608; Abcam; USA) and HIF-2α (1:250; rabbit monoclonal, clone; ab20654; Abcam; USA) in an automatic immunostainer and kept at 4 °C overnight. Then, the immunostainer was put in 37 °C for 60 min before a secondary antibody (ZB-2010; Zhongshan Golden Bridge Biotechnology, China) was added, and then treated with DAB (diaminobenzidine) for 2 min. The sections were investigated using a multiheaded microscope by two investigators who were blinded to the patients’ clinical information.

Stromal staining of HIF expression was graded semiquantitatively as follows: I, no stromal staining; II, little, staining in <10 % of stroma; III, moderate, staining in ≥10 and ≤50 %; IV, strong, staining in >50 % of stroma. For statistical analysis, a final staining score of I or II was combined into the low expression group, and a final staining score of III or IV was combined in the high expression group.

### Protein preparation and Western blotting analysis

Of each sample, 50- to 100-mg tissues from 30 fresh frozen tumors and normal tissues were minced on the ice and sonicated in protein lysis buffer. Then, the protein was collected by centrifuging at 10,000–14,000 rpm for 5 min; total of 30–50 mg of protein from each case was loaded on 10 % SDS polyacrylamide gels and separated by SDS-PAGE, then transferred to PVDF membranes. After blocking with 5 % non-fat milk in TBS-T for 1.5 h, the membranes were treated with the primary antibodies of HIF-1α (1:1000; Abcam; USA), HIF-2α (1:1000; Abcam; USA), and β-actin (1: 3000, Abcam; USA) for 1.5 h at 37 °C, and then kept the membrane at 4 °C overnight. After that, the membranes were incubated with the secondary antibody at an appropriate concentration for 1.5 h. The bands were visualized using an enhanced chemiluminescence detection system.

### Statistical analysis

Statistical analysis was performed by SPSS 17.0 (SPSS Inc., Chicago, IL, USA) statistical software. Continuous variables were expressed as a mean with standard deviation and analyzed using Student’s *t* test (two-tailed). Comparison of the clinicopathological parameters with HIF-1α and HIF-2α expression between the tumor and normal tissues was conducted by the two-tailed chi-square test. The correlation between the expression of HIF-1α and HIF-2α was detected by Spearman rank. *P* value <0.05 was considered to be statistically significant.

## Results

### Immunohistochemistry expression of HIF-1α and HIF-2α in PTC

**Fig. 1:**
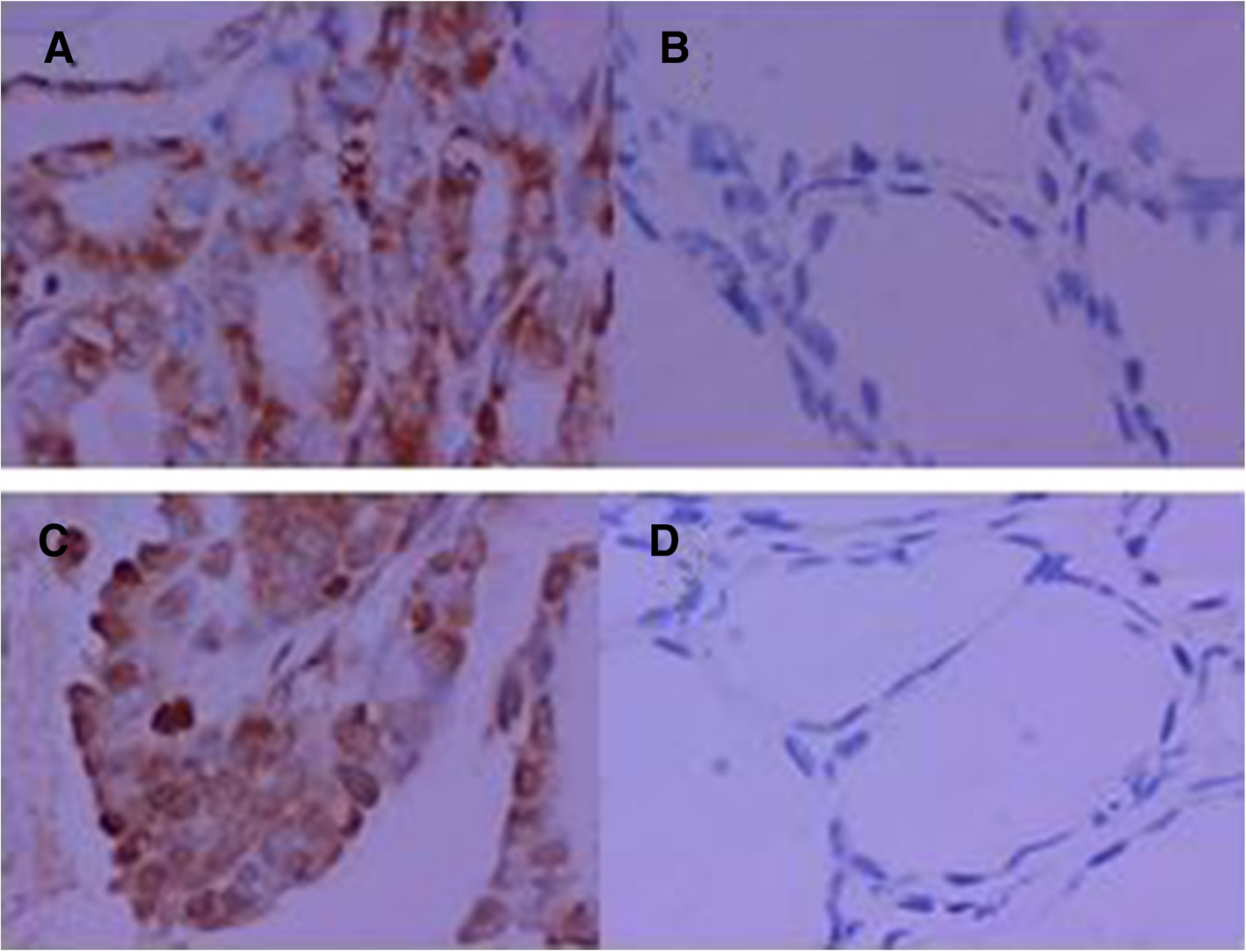
Hypoxia-inducible factor in a papillary thyroid carcinoma. **a** Representative images of HIF-1α in PTC. **b** Representative images of HIF-1α in normal. **c** Representative images of HIF-2α in PTC. **d** Representative images of HIF-2α in normal; ×400

The protein levels of HIF-1α and HIF-2α were first measured by immunohistochemistry (IHC) on paraffin slides of 92 samples of PTC and paired 46 relatively normal tissues. The positive staining of HIF-1α was located in the cytoplasm and the nuclei, but the unequivocal HIF-2α staining was observed mainly in the cytoplasm (Fig. 1). Overexpression of HIF-1α was found in 67 (73 %) of tumor tissues and in 11 (27 %) of the normal group. In consistence, high expression of HIF-2α was also found in 60 (65 %) of tumor tissues, compared with 15 (35 %) of the other group. The differences in HIF-1α and HIF-2α protein expression between PTCs and normal tissues were statistically significant (*P* < 0.01) (Table 1).

**Table 1:** Immunohistochemistry results

Team	HIF-1α		*χ* ^2^	*P* value	HIF-2α		*χ* ^2^	*P* value
	High	Low			High	Low		
Tumor	67	25			60	32		
Normal	11	35	29.86	<0.01	15	31	13.14	<0.01

### Western blot expression of HIF-1α and HIF-2α in PTC

**Fig. 2:**
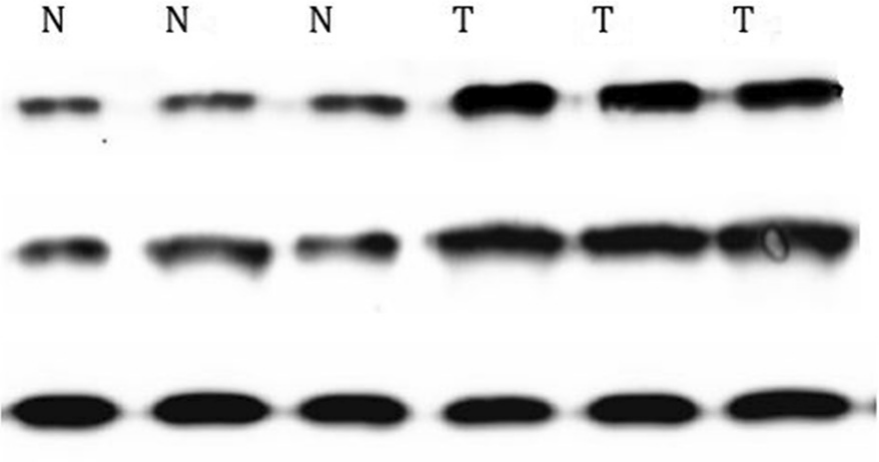
The expression of HIF-1α and HIF-2α protein in PTC tissue and the paired normal tissue was confirmed by Western blotting. Beta-actin was applied as a control. *N* normal, *T* tumor

To explore the expression of HIF-1α and HIF-2α in PTC, Western blot was also applied to detect the protein levels of them in four paired PTC specimens. As shown in Fig. 2, the protein level of HIF-1α in PTC tissues was significantly higher than that in control tissues, the same as HIF-2α.

### Correlations of HIF-1α and HIF-2α protein expression with clinicopathological parameters

Significant correlations of HIF-1α and HIF-2α expression with clinicopathological parameters are listed in Table 2. Regarding the clinical data, no gender-specific distribution was found both in HIF-1α (*P* = 0.223) and HIF-2α (*P* = 0.725).There were also no specific differences in HIF-1α and HIF-2α protein expression between older (>45) and younger (≤45) patients (*P* = 0.882, *P* = 0.675). Both calcification and Hashimoto’s disease of the PTC showed no significant correlation with HIF-1α (*P* = 0.230; *P* = 0.410) and HIF-2α expression (*P* = 0.463; *P* = 0.256).

**Table 2:** Correlation of clinicopathological/morphological parameters and HIF-1α/HIF-2α expression

Clinical Characteristics	PTC	HIF-1α		HIF-2α	
	Total	(+)		(+)	
	*n* (%)	*n* (%)		*n* (%)	
	92 (100 %)	67 (73 %)	*P* value	60 (65 %)	*P* value
Gender					
Female	64 (70)	49 (67)		41 (64)	
Male	28 (30)	18 (64)	0.223	19 (68)	0.725
Age (year)					
≥ 45	49 (53)	36 (74)		31 (63)	
< 45	43 (47)	31 (72)	0.882	29 (67)	0.675
Tumor size (cm)					
≥ 1	42 (40)	30 (71)		32 (76)	
< 1	50 (60)	37 (74)	0.782	28 (56)	0.043*
Lymph node metastasis					
Yes	51 (55)	43 (84)		38 (75)	
No	41 (45)	24 (59)	0.006*	22 (54)	0.037*
Calcification					
Yes	35 (38)	23 (66)		21 (60)	
No	57 (62)	44 (77)	0.230	39 (68)	0.410
Capsular invasion					
Yes	56 (61)	46 (82)		43 (77)	
No	36 (39)	21 (58)	0.012*	17 (47)	0.004*
TNM stage					
I–II	52 (59)	33 (64)		28 (51)	
III–IV	40 (41)	34 (85)	0.021*	32 (77)	0.009*
Hashimoto’s disease					
Yes	62 (67)	47 (76)		38 (61)	
No	30 (33)	20 (67)	0.463	22 (73)	0.256

*HIF* hypoxia-inducible factor

*P < 0.05

Correlation of expression of HIF-1α in PTC tissues was not seen between the larger (≥1) and smaller (<1) tumor size (*P* = 0.782); however, significant differences were observed in HIF-2α expression (*P* = 0.043). Additionally, chi-square test showed a significant association between the presence of lymph node metastasis with HIF-1α (*P* = 0.006) and HIF-2α expression (*P* = 0.037).There was also a statistical significance between capsular invasion with the expression of HIF-1α (*P* = 0.012) and HIF-2α (*P* = 0.004). Generally, patients with unequivocal HIF-1α and HIF-2α staining exhibited a higher incidence of capsular invasion than patients with negative or equivocal HIF-1α and HIF-2α expression. Patients with high HIF-1α staining exhibited a higher TNM stage than patients with low HIF-1α expression (*P* = 0.021), the same as HIF-2α (*P* = 0.009).

### mRNA expression of HIF-1α and HIF-2α in PTCs

**Fig 3:**
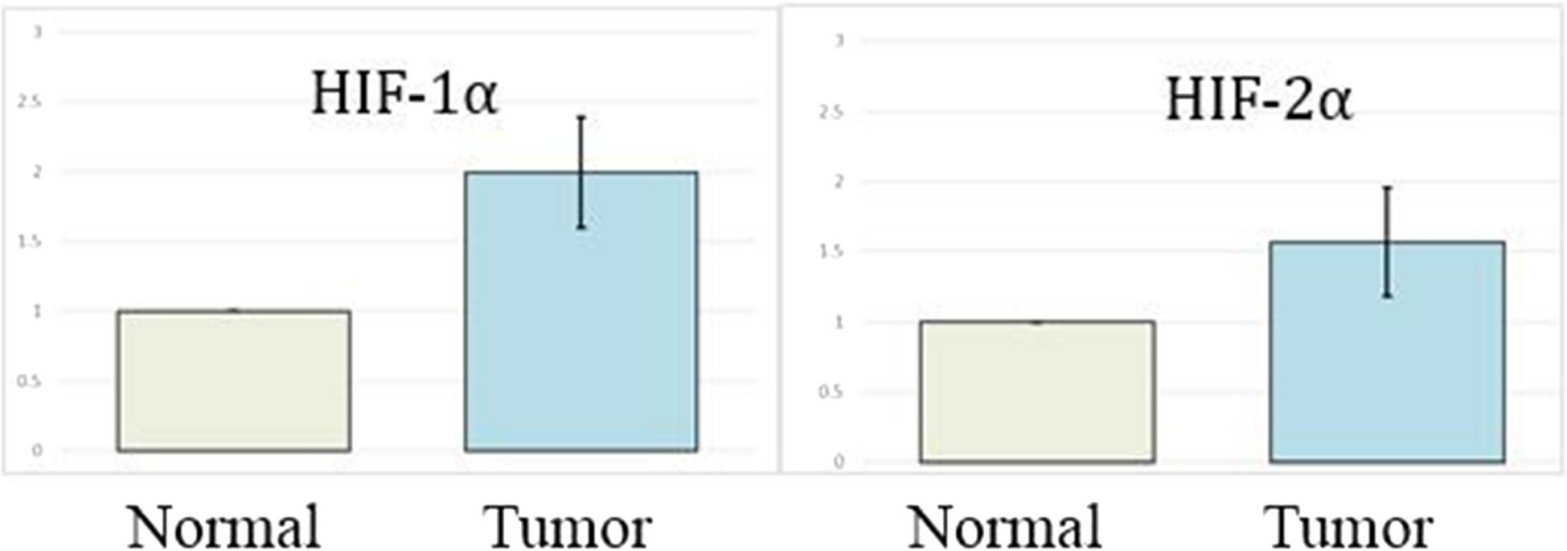
PCR analysis of cDNA reverse-transcribed from mRNA of HIF-1α and HIF-2α in PTC tissues and the normal tissue. GAPDH was used as a control

The mRNA levels of HIF-1α and HIF-2α were showed in Fig. 3. The relative expression of HIF-1α mRNA in PTC was 1.9948 ± 0.3952, and significant differences were observed (*t* = 13.784, *P* < 0.001); the HIF-2α mRNA was 1.5691 ± 0.3889, which is apparently higher than the normal. (*t* = 5.237, *P* < 0.001). According to these results, it is clearly showed that both mRNA and protein expression levels of HIF-1α and HIF-2α were higher in PTCs than the paired normal tissues.

### The correlation of HIF-1α and HIF-2α expression

The correlation of HIF-1α and HIF-2α protein expression was assessed by Spearman rank test. As shown in Table 3. Forty-eight (52 %) PTCs displayed high expression, and 13 (14 %) showed low expression for both HIF-1α and HIF-2α. The correlation between them was statistically significant (*r*_s_ = 0.221, *P* = 0.034).

**Table 3:** Correlation of HIF-1α and HIF-2α protein expression

	HIF-2α		*r*_s_	*P*
HIF-1α	High	Low		
High	48	19	0.221	0.034
Low	12	13		

## Discussion

Hypoxia is a common condition found in a range of solid tumors and has been increasingly recognized to play a key role in different stages of tumor progression [20]. The adaptation of tumor to hypoxia is predominantly regulated by HIF-1α and HIF-2α; while highly homologous, HIF-1α and HIF-2α have unique tissue distributions and play critical but non-overlapping roles in tumor progression [21]. HIF-1α has been implicated as a tumor promoter, while it has been suggested that HIF-2α act as a tumor suppressor gene [22]. To date, studies have observed that HIF-1α and HIF-2α are overexpressed in several human tumors, such as ovarian, esophageal, head and neck [26], breast, prostate, bladder, and oral epithelium [23–26]. Moreover, there have been several studies which respectively showed that HIF-1α [27] and HIF-2α [28] are both upregulated in thyroid carcinoma compared with normal thyroid or benign lesion. However, no studies have examined the expression of HIF-1α and HIF-2α under the same condition and systematically assessed the correlation of their expression with clinicopathological features in PTC simultaneously. In the present study, we observed that an overexpression of HIF-1α and HIF-2α is present in a majority of PTC compared to normal tissues.

As the first HIF family member [29, 30], HIF-1 is an important transcription factor in the development of several tumors. HIF-2α, a gene with high construction gene similarity with HIF-1α, contains 48 % the same basic amino acid sequences, basic helix-loop-helix (bHLH) area with 83 % similarity, and per-amt-sim (PAS) area with 70 % similarity. Recent studies of HIF-2α failed to show consistent results on how HIF-1α and HIF-2α affect the PTC development [31–33]. Our results demonstrated that the positive rate of HIF-1α and HIF-2α in PTCs was 67 (73 %) and 60 (65 %) higher than those in normal thyroid, respectively (*P* < 0.01), which indicated overexpression of HIF-1α and HIF-2α in PTCs, which was confirmed by real-time PCR and Western blot.

The correlation of HIF-1α protein expression with several clinicopathological parameters was also assessed. We found that HIF-1α and HIF-2α protein expression was not associated with gender, age, tumor size, calcification, and Hashimoto’s disease. However, there was a significant correlation of HIF-1α protein expression relative to TNM stage (*P* = 0.021) and LNM (*P* = 0.006). The overexpression of HIF-1α was associated with capsular invasion and high TNM stage and LNM. These results suggested that HIF-1α may play an important role in invasion, metastasis, and progression of PTC. The same with HIF-1α, HIF-2α protein expression was not associated with gender, age, calcification, and Hashimoto’s disease and correlated to TNM stage (*P* = 0.009) and LNM (*P* = 0.037). However, there is a significant association between tumor size and HIF-2α (*P* = 0.035). To date, there is no report about tumor size like ours. It is necessary to further explore the mechanisms. So far, our data confirm the role of HIF-2α in the tumor progression of PTC.

Interestingly, this study demonstrated a significantly positive correlation between HIF-1α and HIF-2α protein expression in PTCs for the first time. HIF-1α expression is positively associated with HIF-2α expression (*P* = 0.034).To date, there was no study could explain this positive correlation in PTCs. It is necessary for us to explore mechanisms underlying this correlation.

Besides HIF-1α and HIF-2α, the other hypoxia family members, HIF-3α for instance, are also involved in cellular responses to hypoxia. HIF-3α has an ability to compete with HIF-1α and HIF-2α to bind with HIF-1β subunits, reducing the levels of HIF-1 and HIF-2, thereby inhibiting the up-regulation of target gene expression induced by HIF-1 and HIF-2 [34]. So, it may also be involved in the development and progression of PTC, and we need to conduct more extensive studies to understand the roles they play in this progress.

This study compares the expression of HIF-1α and HIF-2α in PTCs and normal tissues separately at the level of gene and protein. To our knowledge, it is the first time to demonstrate the significantly positive correlation between HIF-1α and HIF-2α protein expression in PTCs. However, in the present study, it mainly focuses on tissues. Much more extensive studies about mechanisms at the cellular and molecular levels would be needed in the coming future.

## Conclusions

To our knowledge, this is the first study that demonstrates the positive correlation between HIF-1α and HIF-2α in PTCs. High expression of HIF-1α and HIF-2α was associated with high TNM stage and LNM. Consequently, our results provide a possible basis for prediction of LNM and progression in PTC and may provide new therapeutic options targeting hypoxia-associated and hypoxia-regulated proteins. However, the underlying mechanisms still need further exploration, and future studies in larger sets of patients will be necessary to determine the utility of these molecules as biomarkers of tumor diagnosis and prognosis in PTC.

## Abbreviations

ARNT: arylhydrocarbon receptor nuclear translocator
bHLH: basic helix-loop-helix
CT: cycle threshold
GAPDH: glyceraldehyde-3-phosphate dehydrogenase
HIF: hypoxia-inducible factor
IHC: immunohistochemistry
PAS: per-amt-sim
PTC: papillary thyroid carcinoma
